# Health-related quality of life in refugee minors from Syria, Iraq and Afghanistan resettled in Sweden: a nation-wide, cross-sectional study

**DOI:** 10.1007/s00127-021-02050-8

**Published:** 2021-03-22

**Authors:** Øivind Solberg, Mathilde Sengoelge, Charisse M. Johnson-Singh, Marjan Vaez, Anna-Karin Eriksson, Fredrik Saboonchi

**Affiliations:** 1 Division for Implementation and Treatment Research, Norwegian Centre for Violence and Traumatic Stress Studies, Pb. 181 Nydalen, 0409 Oslo, Norway; 2 Division of Insurance Medicine, Department of Clinical Neuroscience, Karolinska Institutet, 171 77 Stockholm, Sweden; 3 Department of Health Science, Swedish Red Cross University College, Stockholm, Sweden; 4 Unit of Mental Health, Children and Youth, The Public Health Agency of Sweden, Solna, Sweden

**Keywords:** Health-related quality of life, KIDSCREEN-27, Refugees, Minors, Resettlement

## Abstract

**Purpose:**

To examine health-related quality of life (HRQoL) in refugee minors resettled in Sweden and compare results to a European reference population, while exploring associations between sociodemographic factors and HRQoL dimensions.

**Methods:**

A cross-sectional, nation-wide study was conducted with a stratified sample of refugee minors ages 12–15 and 16–18 from Afghanistan, Iraq and Syria, resettled in Sweden between 2014 and 2018. HRQoL was measured using KIDSCREEN-27. HRQoL dimension scores of the sample were compared to mean scores of European age and gender-matched reference population. Associations between sociodemographic factors and HRQoL dimensions were investigated with independent *t* tests and ANOVA. A multivariable regression analysis was performed to identify the sociodemographic factors associated with HRQoL.

**Results:**

The questionnaire was sent to 10,000 potential respondents. The response rate was 26%, yielding *n* = 2559 refugee minors (boys 55%, girls 45%) in the study sample. Compared to European references, minors in the present study had significantly lower scores of HRQoL within *psychological wellbeing* and *peers and social support,* whereas levels for *autonomy and parent/guardian relations* and *school environment* were higher. Several sociodemographic factors were significantly associated with all HRQoL dimensions, with those 16–18 years old, having average or poor family economy, and living with an unrelated adult or family reporting lower levels of HRQoL. Minors from Afghanistan had significantly lower scores of HRQoL for all dimensions compared to those from Iraq and Syria.

**Conclusion:**

Refugee minors had significantly lower levels of HRQoL for *psychological wellbeing* and *peers and social support* compared to European references. Future research should further investigate this potential HRQoL gap further.

**Supplementary Information:**

The online version contains supplementary material available at 10.1007/s00127-021-02050-8.

## Introduction

In recent years, children and adolescents have been forced to flee their home countries in increasing numbers due to various and complex reasons and circumstances. These range from conflict and war to natural disasters and poverty. Minors under the age of 18 now represent above one-third of all asylum-seekers worldwide, with 153,000 reported as unaccompanied in 2019 [1]. There are also age- and gender-specific threats involved, including forced recruitment, sexual exploitation and being forced to leave parents [2–4]. In 2019, one-third of asylum applicants to Europe were minors aged less than 18 years [5]. During the increase in forced migration to Europe in 2015, Sweden accepted more refugees, including unaccompanied refugee minors, than any other European country. In total, 70,000 children and adolescents sought asylum in Sweden in 2015, and approximately half of these applicants were unaccompanied minors from Afghanistan, Iraq and Syria [6]. Refugee minors have continued to apply for asylum in Sweden since 2015, albeit in smaller numbers [7] . The term “unaccompanied” refers here to the European Union (EU) asylum acquis’ definition of an unaccompanied minor refugee, being a non-EU national or stateless person under the age of 18 who arrives on the territory of the EU unaccompanied by an adult guardian responsible for him/her.

Previous research has shown that minors are the most vulnerable refugees, with an increased risk of mental ill health, and that unaccompanied minors constitute an especially high-risk group as they have to cope with insecure visa status, temporary residence and increased vulnerability to traffickers and exploitation, due to the lack of protection or support from family members/guardians [8]. Systematic reviews among refugees 5–17 years of age have estimated the prevalence of post-traumatic stress disorder (PTSD) between 7 and 54%, anxiety 33–50% and depression 3–30% [9–13]. In Sweden specifically, previous studies have found similar findings of ill health in refugee populations newly resettled [14–17]. A systematic review conducted in Europe found that unaccompanied female refugee minors had a higher vulnerability to post-traumatic and depressive symptoms compared to their male counterparts [18] and a recent review identified the number of stressful live events to be the risk factor for mental health problems in unaccompanied refugee minors, while a stable environment and social support were protective factors [19]. Two reviews and meta-analyses highlight an alarming heterogeneity in the methods used and low generalizability of findings [20, 21]. Challenges related to reaching this “hard-to-reach” population have also led to the widespread use of convenience sampling rather than a probability sampling approach as part of a more scientifically robust survey methodology, in turn decreasing the generalizability of findings [22–24]. Contextual drivers for mental ill health seem to be pre-, peri- and post-migratory stressors, including accumulated stress due to trauma, living conditions, socioeconomic strain, social isolation, discrimination, and difficulties in communicating and absorbing information [25–27].

Still, it is also important to note that refugee minors are a heterogeneous group in terms of flight experiences, status on arrival (accompanied vs. unaccompanied), age, gender, family constellation, country of origin/birth and the conditions they live under may vary to a large degree [28]. Previous research has, to some degree, neglected this fact and tended to group “refugees” together as a homogenous group, in turn implying that they are likely to be in a similar psychosocial situation despite, e.g., the range of different pre- and peri-flight conditions they might have experienced [29]. Here, vulnerability to ill health and access to resources may vary, and unaccompanied minors have been shown to be particularly at risk [30, 31]. Health and conditions of growth during childhood can impact future social development and health throughout the life course [32]. Therefore, it is important to understand how young refugees from different departures and circumstances experience their health-related quality of life (HRQoL) after resettling in a host country. Furthermore, it is crucial to identify the sociodemographic factors that increase or diminish HRQoL during adolescence, as it is a critical period in the developmental stage that may affect health throughout life [33]. As adolescence is a stage when young people may modify or alter the pathways to adult health or illness [34], a window of opportunity also arises here for interventions targeting health and wellbeing.

Although previous research focusing on forced migration, mental health, and psychosocial adjustment in young refugees and asylum-seekers has indicated a heightened risk of mental ill health, no study to date has examined health-related quality of life (HRQoL) within this population. Gaining an understanding of the factors that contribute to adolescent refugees’ health and wellbeing is considered necessary to promote positive development [35]. It is, therefore, pertinent that the research field go beyond identifying medical conditions in adolescent refugees and move towards gaining insight into their optimal functioning from a more ecological, holistic perspective [36].

Therefore, the aim of the present study was to assess HRQoL in refugee minors who were granted residency in Sweden and compare findings to a European reference population, while exploring associations between sociodemographic factors and HRQoL dimensions.

## Methods

### Participants and procedure

The source population for the present study consisted of all refugee minors aged 12–18 years from Afghanistan, Iraq and Syria who were granted residency in Sweden and accepted into a municipality between 2014 and 2018 (*N* = 25,584). This source population was identified through the Total Population Register held by Statistics Sweden covering every individual that resides in Sweden on a permanent basis. The source population was stratified by country of birth, gender and age group (12–15 years, *n* = 13,271; and 16–18 years, *n* = 12,313) for a total of 12 strata, and 10,000 individuals were selected from this stratified sampling frame.

An invitation to participate, including a detailed information letter and a questionnaire in the appropriate language (Swedish and either Arabic or Dari/Farsi), was sent by post with a pre-paid return envelope enclosed to the parent/guardian of minors aged 12–15. Potential respondents aged 16–18 received the same invitation, directly addressed to themselves. A reminder was sent to non-responders after 4 weeks, followed by a second reminder after a total of 8 weeks, again containing the questionnaire. The act of completing the questionnaire and sending it in was regarded as an informed consent. Data collection occurred between June and September 2018. A total of 2559 boys and girls responded, giving a response rate of 26%.

### Questionnaire

For questions/questionnaire text not already available in Arabic and Dari/Farsi, a standard double-blind translation and back-translation procedure was used [37] for linguistic adaptation of the present study’s questionnaire. As an additional precaution, in 2018 after ethical approval was obtained, 15 Arabic and Farsi-speaking children filled-out the full questionnaire and then underwent cognitive interviews with verbal probing to identify any ambiguities in the information provided or the questions themselves [38–40]. In cases where difficulties were detected, the questionnaire text was reviewed together with a professional interpreter as well as native speaking community members. Following these procedures, minor adjustments in wording and phrasing were performed. The KIDSCREEN-27 measurement scale was not included in this process, since this scale was available in both Swedish, Arabic and Dari/Farsi from the KIDSCREEN group, at KIDSCREEN.org.

### Measurements

#### Health-related quality of life (HRQoL)

HRQoL was measured using validated and certified Swedish, Arabic and Dari/Farsi versions of KIDSCREEN-27. The KIDSCREEN-27 instrument was developed by Ravens-Sieberer [41] for children and adolescents aged 8–18 years and designed to measure children’s and adolescents’ subjective health and wellbeing. It has demonstrated robust psychometric properties [42] and cross-cultural validity [43]. The instrument consists of 27 questions that reflect 5 HRQoL dimensions: *physical wellbeing* (five questions), *psychological wellbeing* (seven questions), *autonomy and parent relations* (seven questions, here adapted to include the relationship with guardians/caregivers given the context), *peers and social support* (four questions) and *school environment* (four questions). In accordance with the KIDSCREEN scoring syntax, each question is scored on a five-point scale (1 = “not at all”, 2 = “a little”, 3 = “reasonably”, 4 = “very” and 5 = “very much”). The responses within each dimension are summed. A standardized scoring algorithm is used to assign a Rasch person parameter to each sum score. Individual parameters are then converted to *t*-scores for each dimension and scaled to a mean of 50 and a standard deviation of 10. Higher *t*-scores indicate higher levels of HRQoL.

#### Sociodemographic characteristics

Sociodemographic variables include age, gender, country of birth, status on arrival, living situation and family economy. Information on age, gender and country of birth were obtained from Statistics Sweden’s Total Population Register and used in categorical form, with age categories 12–15 and 16–18, gender categories as boy or girl, and country of birth categories as Afghanistan, Iraq or Syria.

The variable ‘status on arrival’ was provided by Statistics Sweden’s STATIV database and was used to define a participant as a refugee minor, unaccompanied or accompanied. In concord with EU’s asylum acquis’ definition, the STATIV database states that “an unaccompanied refugee minor is defined as a refugee under 18 years of age who arrives in Sweden without a parent or other legal guardian” [44].

All participants were asked which adults they lived with. There were eight possible answers: (1) *I live with both my mother and father*; (2) *My parents do not live together and I live with my mother most/all of the time*; (3) *My parents do not live together and I live with my father most/all of the time*; (4) *My parents do not live together and I live equally with my mother and father*; (5) *I live with one or more adult relatives*; (6) *I live with an unrelated adult/adults*; (7) *I live in a family home or equivalent*; and (8) *I live in an HVB-home*. An HVB-home is government-funded institution/home where unaccompanied minors temporarily live and receive basic support. The eight categories were aggregated into three categories: living with parent(s)/or other relative(s) (1–4); living with other adult (s)/or in family home (5–7); and living in residential care (8). Information on family economy was derived from the question “How do you think the family you live with are economically?” with five response alternatives ranging from “very good” to “not at all good”. These responses were trichotomized as follows: “good”, “average”, and “poor”.

### Statistical analysis

Descriptive statistics were performed to summarize the sociodemographic profile of the survey participants (*n* = 2559) in relation to the sample frame (*n* = 10,000). Given the sensitivity of Pearson’s Chi-square test to large sample sizes, Cohen’s *h* was used to assess the extent of differences in sociodemographic characteristics between respondents and non-respondents, with *h* = 0.20 denoting a small effect size and *h* = 0.50 and *h* = 0.80 indicating medium and large effect sizes, respectively [45]. Mean *t*-scores for the five HRQoL dimensions were estimated for the whole sample and then separately for boys and girls. Two-sample *t* tests were used to compare the sample in the present study with age- and gender-matched European scores as a reference [41].

To examine whether the differences in HRQoL were significant between groups within each sociodemographic variable, a series of independent two-sample *t* tests and one-way analysis of variance (ANOVA) with post hoc tests were performed. Where homogeneity of variance was lacking, Welch’s *t* test was used for the dichotomous variables and the Tamhane post hoc test for the trichotomous variables. Multivariable linear regression was used to investigate the association of sociodemographic factors with each HRQoL dimension in two models. Model 1 adjusted for age and gender and model 2 additionally adjusted for status on arrival, living situation, and family economy. Country of birth was not included due to the high collinearity with other variables, primarily status on arrival. The adjusted *R*^2^ was reported to demonstrate the explained variance of each model. Assumptions of normally distributed residuals, homoscedasticity, and no multicollinearity were tested and met for the fully adjusted models of each outcome (model 2). All analyses were performed using SPSS Version 25.

### Ethics

The study was reviewed and approved by the Stockholm Regional Ethical Review Board (approval number 2018/456-31/5). Statistics Sweden was responsible for conducting the survey. Data were analyzed anonymously. Enclosed with the questionnaire was an informed consent form explicitly stating that participation was voluntary and submission of the questionnaire implied consent. The survey was sent by post, data were de-identified by Statistics Sweden and only a small research team had access to the anonymized data.

## Results

### Sociodemographic characteristics

The distribution of sociodemographic characteristics for the sample frame (where available) and study population are shown in Table 1. Over half of the 2559 respondents were boys (55%) and between 12 and 15 years old (56%). One in five (19%) respondents immigrated to Sweden unaccompanied. A large majority (88%) stated that they live with their parent(s) or relative(s), compared to 7% living with an unrelated adult or family, and 5% in residential care.

**Table 1.** Sociodemographic characteristics of the sample frame and study respondents

	Sample frame (*N* = 10,000)		Respondents (*N* = 2559)		Cohen’s *h*^a^ (respondents vs. non-respondents)
	*n*	%	*n*	%	
Age					
12–15	4929	49.3	1430	55.9	0.178
16–18	5071	50.7	1129	44.1	− 0.178
Gender					
Boy	5707	57.1	1402	54.8	− 0.062
Girl	4293	42.9	1157	45.2	0.062
Country of birth					
Afghanistan	3038	30.4	741	29.0	− 0.041
Iraq	1930	19.3	447	17.5	− 0.062
Syria	5032	50.3	1371	53.6	0.088
Status on arrival					
Accompanied	7503	75.0	2079	81.2	0.198
Unaccompanied	2497	25.0	480	18.8	− 0.198
Living situation					
With parent(s) or other relative(s)	(NA)	(NA)	2132	87.7	(NA)
With other adult(s) or in family home	(NA)	(NA)	178	7.3	(NA)
In residential care	(NA)	(NA)	120	4.9	(NA)
Family economy					
Good	(NA)	(NA)	961	38.7	(NA)
Average	(NA)	(NA)	1066	42.9	(NA)
Poor	(NA)	(NA)	458	18.4	(NA)

^a^Cohen’s *h* estimates an effect size for the differences between respondents and non-respondents. Differences are considered small if *h* = 0.2, medium if *h* = 0.5, or large if *h* = 0.8.

Over one-third (39%) of respondents stated that their families/those with whom they live are economically doing good, whereas 43% indicated that they had average family economy and 18% reported poor family economy. Cohen’s *h* calculations determined that all differences in sociodemographic characteristics between respondents and non-respondents could be considered negligible to small (−0.2 ≤ *h* ≤ 0.2).

### HRQoL compared to European reference population

Table 2 presents age and gender comparisons for each HRQoL dimension score for refugee minors compared to the European reference population. Overall, respondents in the present study reported significantly higher HRQoL compared to European minors for two dimensions, *relation to parents and autonomy* and *school environment,* and significantly lower levels for two dimensions, *psychological wellbeing* and *peers and social support*. The same pattern was observed when comparing refugee boys and girls to their respective European counterparts, except for *psychological wellbeing* in girls, which was not significant. Differences in *physical wellbeing* were also not significant between the refugee and European cohorts. See Online Resource 2 for a visualization of the sum score scale comparison.

**Table 2:** Age and gender comparison of KIDSCREEN-27 dimension scores for refugees compared to the European reference population

KIDSCREEN-27 dimensions	Total					Gender									
						Girls					Boys				
	Refugee		EU reference^a^		*p* value	Refugee		EU reference^a^		*p* value	Refugee		EU reference^a^		*p* value
	*N*	Mean (SD)	*N*	Mean (SD)		*n*	Mean (SD)	*n*	Mean (SD)		*n*	Mean (SD)	*n*	Mean (SD)	
Physical wellbeing	2535	49.2 (11.6)	15,239	48.6 (9.6)	**0.014**	1150	47.4 (11.3)	8077	46.8 (9.2)	0.085	1385	50.8 (11.5)	7139	50.5 (9.8)	**0.364**
Psychological wellbeing	2507	47.2 (12.9)	15,323	48.8 (9.8)	**< 0.000**	1135	46.9 (13.1)	8142	47.3 (9.6)	0.322	1372	47.4 (12.7)	7156	50.6 (9.7)	**< 0.000**
Autonomy and parent relations	2488	54.4 (12.0)	15,135	49.4 (9.8)	**< 0.000**	1137	54.7 (12.0)	8045	48.5 (9.8)	**< 0.000**	1351	54.1 (12.0)	7067	50.4 (9.8)	**< 0.000**
Peers and social support	2520	46.2 (12.3)	15,372	49.6(10.0)	**< 0.000**	1144	45.7 (12.9)	8161	50.1 (10.1)	**< 0.000**	1376	46.6 (11.7)	7188	49.1 (9.9)	**< 0.000**
School environment	2512	52.9 (9.5)	15,255	48.4 (9.4)	**< 0.000**	1140	53.2 (9.4)	8102	48.5 (9.2)	**< 0.000**	1372	52.6 (9.6)	7130	48.3 (9.7)	** < 0.000**

Bold *p*-values denote statistical significance at *α* = 0.05

^a^Reference values are based on KIDSCREEN-27 with a 12- to 18-year-old reference sample [41]

Table 3 shows the differences in mean HRQoL scores between sociodemographic factors. Respondents aged 12–15 years scored significantly higher HRQoL for all dimensions, compared to those 16–18 years old. Boys scored significantly higher for *physical wellbeing* than girls. Respondents who immigrated to Sweden unaccompanied scored significantly lower HRQoL for all dimensions, compared to those who came to Sweden accompanied. The results in Table 3 also show that there were significant differences between sociodemographic factors regarding living situation, family economy, and country of birth. Post hoc test results (Online Resource 1) demonstrated that respondents who lived with a parent(s)/relative(s) scored significantly higher HRQoL for all dimensions than those who lived with another adult/family or in a residential home. Furthermore, those who lived with other adults/family scored significantly higher HRQoL with regards to *psychological wellbeing, autonomy and parent relations*, and *peers and social support* dimensions, compared to those living in residential care. Respondents who reported that their family economy was good had the highest quality of life for all dimensions, followed by average and then poor family economy. These differences were statistically significant for all categories. Respondents born in Afghanistan scored significantly lower HRQoL for all dimensions, compared to those from Iraq and Syria.

**Table 3:** Association between sociodemographic characteristics and health-related quality of life dimensions in refugee minors

	Physical wellbeing		Psychological wellbeing		Autonomy and parent relations		Peers and social support		School environment	
	Mean (SD)	*p* value	Mean (SD)	*p* value	Mean (SD)	*p* value	Mean (SD)	*p* value	Mean (SD)	*p* value
Age										
12–15	52.0 (10.9)	**< 0.000**^a^	51.0 (12.2)	**< 0.000**^a^	57.5 (11.1)	**< 0.000**^a^	48.2 (11.5)	**< 0.000**^a^	54.7 (9.2)	**< 0.000**^a^
16–18	45.8 (11.5)		42.4 (12.2)		50.4 (12.0)		43.7 (12.7)		50.6 (9.4)	
Gender										
Boy	50.8 (11.5)	**< 0.000**^a^	47.4 (12.7)	0.380^a^	54.1 (12.0)	0.223^a^	46.6 (11.7)	0.078^a^	52.6 (9.6)	0.137^a^
Girl	47.4 (11.3)		46.9 (13.1)		54.7 (12.0)		45.7 (12.9)		53.2 (9.4)	
Status on arrival										
Accompanied	50.0 (11.6)	**< 0.000**^a^	48.6 (12.7)	**< 0.000**^a^	55.7 (11.7)	**< 0.000**^a^	46.9 (12.4)	**< 0.000**^a^	53.7 (9.3)	**< 0.000**^a^
Unaccompanied	46.2 (10.7)		41.1 (12.0)		48.2 (11.6)		43.5 (11.3)		49.3 (9.7)	
Living situation										
With parent(s) or other relative(s)	50.0 (11.7)	**< 0.000**^b^	48.5 (12.8)	**< 0.000**^b^	55.6 (11.6)	**< 0.000**^b^	46.8 (12.3)	**< 0.000**^b^	53.7 (9.3)	**< 0.000**^b^
With other adult(s) or in family home	45.0 (9.4)		40.7 (11.2)		48.5 (10.8)		44.3 (11.5)		49.2 (8.9)	
In residential care	43.9 (9.5)		37.0 (10.5)		41.5 (11.3)		40.6 (10.5)		47.0 (9.7)	
Family economy										
Good	51.8 (11.0)	**< 0.000**^b^	51.6 (12.7)	**< 0.000**^b^	58.3 (11.7)	**< 0.000**^b^	49.8 (11.4)	**< 0.000**^b^	55.1 (9.3)	**< 0.000**^b^
Average	49.0 (11.2)		46.7 (12.3)		54.5 (11.2)		45.6 (12.0)		52.9 (9.2)	
Poor	45.0 (12.3)		39.7 (11.0)		46.4 (10.4)		40.9 (12.3)		48.6 (9.5)	
Country of birth										
Afghanistan	46.1 (11.0)	**< 0.000**^b^	42.4 (12.2)	**< 0.000**^b^	49.1 (11.2)	** < 0.000**^b^	42.2 (12.1)	**< 0.000**^b^	50.0 (9.7)	**< 0.000**^b^
Iraq	50.6 (11.9)		49.6 (13.0)		56.8 (12.1)		47.7 (13.0)		54.5 (9.3)	
Syria	50.6 (11.4)		48.9 (12.6)		56.4 (11.6)		47.9 (11.6)		53.9 (9.2)	

^a^Two-sample independent *t* tests, *α* = 0.05, significant *p*-values in bold

^b^One-way ANOVA with post hoc tests, *α* = 0.05, significant *p*-values in bold

Table 4 shows the multivariable associations between sociodemographic factors and the five HRQoL dimensions presented in two models. Respondents in the older age group (16–18 years old) scored significantly lower HRQoL for all five dimensions than younger respondents (12–15 years old) in model 1. The inclusion of additional sociodemographic factors in model 2 slightly attenuated the strength of these associations, but they remained significant. There was also a significant negative association for girls compared to boys with regards to *physical wellbeing* in model 1, which increased after inclusion of other sociodemographic characteristics in model 2. Model 1 showed no significant associations for girls compared to boys with regards to *psychological wellbeing*, *autonomy and parent relations* or *peers and social support,* whereas there was a significant negative association between gender and these dimensions in model 2.

**Table 4:** Multivariable associations between sociodemographic factors and five KIDSCREEN-27 HRQoL dimensions

	Physical wellbeing		Psychological wellbeing		Autonomy and parent relations		Peers and social support		School environment	
	Model 1	Model 2	Model 1	Model 2	Model 1	Model 2	Model 1	Model 2	Model 1	Model 2
	Coefficient (SE)	Coefficient (SE)	Coefficient (SE)	Coefficient (SE)	Coefficient (SE)	Coefficient (SE)	Coefficient (SE)	Coefficient (SE)	Coefficient (SE)	Coefficient (SE)
Age										
12–15	Ref	Ref	Ref	Ref	Ref	Ref	Ref	Ref	Ref	Ref
16–18	− 6.106*** (0.441)	− 4.588*** (0.468)	− 8.638*** (0.489)	− 6.175*** (0.500)	− 7.110*** (0.465)	− 4.660*** (0.459)	− 4.563*** (0.483)	− 3.116*** (0.507)	− 4.104*** (0.374)	− 2.679*** (0.392)
Gender										
Boy	Ref	Ref	Ref	Ref	Ref	Ref	Ref	Ref	Ref	Ref
Girl	− 3.233*** (0.440)	− 4.313*** (0.463)	− 0.205 (0.488)	− 1.884*** (0.494)	0.820 (0.463)	− 0.890* (0.453)	− 0.738 (0.482)	− 1.463** (0.501)	0.714 (0.373)	− 0.263 (0.388)
Status on arrival										
Accompanied		Ref		Ref		Ref		Ref		Ref
Unaccompanied		− 0.267 (0.898)		− 2.245* (0.966)		− 2.090* (0.895)		− 1.003 (0.982)		− 1.540* (0.751)
Living situation										
With parent(s) or relative(s)		Ref		Ref		Ref		Ref		Ref
In a family home or other adult		− 6.634*** (1.194)		− 7.211*** (1.275)		− 6.635*** (1.181)		− 3.083* (1.304)		− 3.638*** (0.997)
HBV-home		− 3.744** (1.412)		− 4.672** (1.508)		− 8.428*** (1.423)		− 2.395 (1.532)		− 3.118** (1.189)
Family economy										
Good		Ref		Ref		Ref		Ref		Ref
Average		− 3.015*** (0.497)		− 5.061*** (0.529)		− 4.075*** (0.487)		− 4.202*** (0.537)		− 2.360*** (0.416)
Poor		− 6.407*** (0.646)		− 11.162*** (0.691)		− 11.241*** (0.635)		− 8.678*** (0.699)		− 6.041*** (0.540)
Adjusted *R*^2^	0.090	0.140	0.110	0.219	0.086	0.231	0.035	0.099	0.046	0.107

Model 1 included age and gender; model 2 additionally included accompaniment status, living situation, and family economy

*B* unstandardised coefficient

*p value < 0.05; **p value < 0.01; ***p value < 0.0

All remaining sociodemographic characteristics in model 2 showed significantly negative associations with *psychological wellbeing, autonomy and parent relations* and *school environment*—being unaccompanied versus accompanied; living with a family/other adult or in a residential home compared with parent(s)/relative(s); and having average or poor family economy compared to good. The same pattern was demonstrated for *physical wellbeing* and *peers and social support*, except that being unaccompanied was not significantly associated with HRQoL for these dimensions, nor was living in a residential home for the former dimension.

The adjusted R^2^ showed that age and gender accounted for between 3.5 and 11.0% of the variance in model 1, for each dimension. Adding status on arrival, living situation, and family economy, for each dimension in model 2, more than doubled the percentage of the variance explained by the model, ranging between 9.9 and 23.1% (except for *physical wellbeing* which explained 30% of the variance).

## Discussion

Although previous research focusing on forced migration, mental health, and psychosocial adjustment in young refugees has indicated a heightened risk of mental ill health, no study to date has examined health-related quality of life (HRQoL) within this population. The aim of the present study was, therefore, to assess HRQoL in refugee minors resettled in Sweden and compare findings to a European age- and gender-matched reference population, in addition to exploring the potential associations between sociodemographic factors and HRQoL scores.

Refugee minors in the present study had significantly lower HRQoL levels, compared to European youth of the same age range with regards to *psychological wellbeing* and *peers and social support.* Conversely, refugee minors scored significantly higher on *autonomy* *and parent relations* and *school environment* compared to their European counterparts. Below, we explore this finding in more detail.

The general gender pattern of girls reporting lower HRQoL in the EU reference population is coherent with the pattern seen among refugee minors in the present study, though the gap was smaller and there was some variability depending on the specific dimensions and when adjusting for socioeconomic characteristics. In a study of young Syrian refugees resettled in Norway similar results were found, where being female was associated with a lower HRQoL Index and *psychological wellbeing* scores [46]. A gradual decrease in mean scores for all dimensions of HRQoL with increasing age in the Norwegian sample was also comparable to our finding that minors aged 12–15 had significantly higher HRQoL scores compared to minors aged 16–18.

Moreover, minors who fled to Sweden unaccompanied scored significantly lower HRQoL scores compared to minors who came to Sweden accompanied. In the fully adjusted model, scores were significantly lower within the dimensions of *psychological wellbeing, autonomy and parent relations*, and *school environment,* possibly reflecting the psychological strain of traveling without parents/guardian(s), and/or the lack of contact with parents/guardians that unaccompanied minors face, both during their flight and after resettlement, as well as a limited potential for schools and teachers to offer support that might compensate for these experiences. The potential negative impact of minors’ flight to a host country may also be exacerbated if they remain alone after resettlement. Minors who lived with parent(s)/relative(s) scored significantly higher on HRQoL within all dimensions compared to minors living with another adult/family or in a residential home. This finding is in line with a recent systematic review that demonstrated that the duality of both risk and protection inherent in the family composition (parental mental health problems and impaired parenting) were risk factors for mental health problems in young refugees, while family cohesion was protective [47].

The minors who reported that their family economy was good had the highest quality of life for all dimensions. This is in line with a study of unaccompanied and accompanied refugee minors resettled in Germany showing that having more everyday resources was a major predictor of lower levels of psychological distress [48]. Finally, minors born in Afghanistan scored significantly lower HRQoL for all dimensions compared to those from Iraq and Syria. A possible explanation for this may be that refugee minors from Afghanistan were to a larger extent unaccompanied compared to those from Iraq and Syria [49]. Another is that the majority lived without a parent or relative, which places them at significantly higher risk for mental health problems [50]. An earlier study of young Afghan adults resettled in Seattle found Pashtun ethnicity, amount of English spoken by their mothers and total number of traumatic events experienced significantly predicted PTSD and major depression [51]. Unaccompanied Afghan minors in the United Kingdom reported high rates of separation from their family (61%) and losing loved ones (80%), indicating the high stress burden [52]. It is important to note here that the refugee minors in this study had been granted a residence permit in Sweden, which means that they knew they were allowed to stay in the country, at least temporarily, and were most likely not concerned with the issues of possible deportation. It is also important to note that previous studies have shown that a long asylum processes and its inherent uncertainty is associated with an increased risk of both physical and mental ill health in young unaccompanied asylum-seekers compared to host populations [30, 53–55]. Some also face detention due to their age being disputed, causing additional stress associated with high rates of psychiatric disorders [48]. Thus, HRQoL in populations of refugee minors seeking asylum could reveal even lower levels of HRQoL. Future research should therefore also explore HRQoL levels within the setting of a reception or detention center for minors seeking asylum to investigate this possibility further.

Together, these results add to the literature on refugee minors’ mental health in revealing that minors overall report lower levels of HRQoL, not only within the dimension of *psychological wellbeing* [46], but also within dimensions of *peers and social support,* while at the same time scoring higher within the dimensions of on *autonomy and parent relations* and *school environment*. The wider scope inherent in the HRQoL measure, therefore, reveals a more complex picture. This broadens our understanding of the psychosocial situation these minors face as newly resettled refugees in a European host country, compared to European youth of similar age and gender. In a related vein, the finding that refugee minors living with other adult(s) or in family homes did not differ in HRQoL scores within the dimensions of *physical wellbeing* and *school environment* compared to minors living in residential care, might allude to the fact that residential care in Sweden provides young refugees with support within these dimensions of HRQoL. In residential care in Sweden, social workers facilitate access to peers and social support through tailored school and leisure activities, while at the same time ensuring adequate health care and psychological support [56, 57]. Here again, the wider scope inherent in the HRQoL measure might indirectly reveal the need for a broader conceptualization of interventions for this group of children, going beyond the standard therapy-based psychological approach. In fact, a recent systematic review and meta-analysis found that psychosocial interventions with a trauma focus provided significant beneficial effects on PTSD, depression and anxiety outcomes in refugees and asylum-seekers [58]. Psychotherapeutic interventions for young unaccompanied refugees were also found to include the development of coping strategies for acculturation-related stressors, in turn improving self-esteem and rebuilding identity [59, 60]. Multimodal interventions that address the preflight, flight and resettlement stressors of exposure to previous traumatic events, adjustment to new environments, linguistic and legal assistance, family and parenting support, and school-based interventions have also shown promise [61, 62]. Thus, the complexity underlying mental ill health and low levels of HRQoL in young refugees may warrant a shift towards a more ecological, culturally sensitive approach to improve the wellbeing of refugee children and their families [63].

Finally, almost one-fifth (18%) of the minors in the present study reported a poor family economic situation. This finding is especially worth noting since minors who reported that their family economy was good had the highest quality of life for all HRQoL dimensions. Indeed, previous research has shown that low-income families are at increased risk on a number of health-related outcomes (all-cause mortality, suicide attempt, alcohol and drug misuse) as well as for comparatively low educational attainment and higher need for social assistance in young adulthood [64].

### Strengths and limitations

To our knowledge, this one of the few studies utilizing KIDSCREEN-27 to assess HRQoL within a population of refugee minors resettled in a high-income host country. Major strengths include the randomized, large sample frame incorporating refugee minors from Afghanistan, Syria and Iraq and the cross-cultural validation of the measures used. Due to KIDSCREEN being a reliable, valid, sensitive, and conceptually/linguistically appropriate HRQoL measure [43], we were able to compare HRQoL levels within the study population to a European age and gender-matched reference population. Yet, several limitations need mentioning.

As less than a quarter of invited refugee minors participated in the present study, selection bias may have influenced the results, and therefore, may not be representative of all refugee minors in Sweden. If non-participation in the study was positively associated with poorer written language proficiency and/or poorer mental health as some evidence suggests [65], the HRQoL among refugee minors may be overestimated. Furthermore, selection bias may also have affected the association between status on arrival (unaccompanied vs. accompanied) and HRQoL, although exposure-outcome associations are generally considered to be quite robust to nonresponse [66, 67]. Children with a refugee background are sometimes referred to as a ‘hard to reach population’ within research contexts as they are generally difficult to contact or decline to participate in large-scale questionnaires [68]. Thus, our response rate and total sample size may be considered quite high when considering the challenges involved in reaching this population, especially children as young as age 12. Still, it is unknown whether the response rates may have differed between guardians receiving the questionnaire on behalf of young minors 12–15 years old, compared to minors 16–18 years old receiving the letter themselves. This may have a potential correlation with quality of life, but one cannot predict in what direction. An assessment of the economic situation in the children’s home country was also not obtained, and it is, therefore, uncertain whether the children’s subjective perception of their current economic situation is colored by a possible discrepancy between their previous and current economic situation.

Finally, since the categorization of refugee minors as unaccompanied vs. accompanied was based on a pre-existing variable obtained from Statistics Sweden, defined as “traveling without a parent or other legal guardian”, it is important to note that “unaccompanied” in this study might include refugee minors that traveled with friends or other family members (i.e., not alone), but without a parent/guardian present. This could underestimate the impact of being unaccompanied on HRQoL scores, with the impact being more significant than what our results show for minors who actually traveled alone, without friends, family or guardian(s).

## Concluding remarks and implications

The present study identifies different gaps in the HRQoL between refugee minors and their European counterparts. Together, the results add to the literature on refugee minors’ mental health in revealing that minors overall report lower levels of HRQoL, not only within the dimension of *psychological wellbeing*, but also within dimensions of *peers and social support,* while at the same time, scoring higher within the dimensions of on *autonomy and parent relations* and *school environment*. The wider scope inherent in the HRQoL measure, therefore, reveals a complex picture, with minors from Afghanistan appearing especially vulnerable. Multimodal interventions addressing this *psychological wellbeing* and social support are, therefore, warranted at the individual and societal level, specifically interventions that can improve HRQoL among adolescents 16–18 years of age. As adolescence is a key developmental stage, a window of opportunity exists where tailored interventions can alter health-trajectories of refugee minors for the better, when it is needed the most.

## Supplementary Information

Below is the link to the electronic supplementary material.

Supplementary file1 (DOCX 1273 KB)
