# Supplementary material for: Health-related quality of life in refugee minors from Syria, Iraq and Afghanistan resettled in Sweden: a nation-wide, cross-sectional study
Source: Soc Psychiatry Psychiatr Epidemiol. 2021 Mar 22;57(2):255–66. doi: 10.1007/s00127-021-02050-8 (PMC8784357; doi:10.1007/s00127-021-02050-8)
Supplement: Supplementary file 1 — Supplementary file1 (DOCX 1273 KB) [file 127_2021_2050_MOESM1_ESM.docx]

SUPPLEMENTARY INFORMATION

Online Resource 1. ANOVA post-hoc tests

| **Living situation - test proves homogeneity of variance for only PH and SS, so use Tukey post-hoc for those, and Tamhane for PW, PA, SC:** | | | | | | | | | | | |  |  |  |  |  |  |  |  |
| --- | --- | --- | --- | --- | --- | --- | --- | --- | --- | --- | --- | --- | --- | --- | --- | --- | --- | --- | --- |
|  |  | Physical wellbeing (PH) | | | | | Psychological wellbeing (PW) | | | | Parent relations and autonomy (PA) | | | Social support and peers (SS) | | | School environment (SC) | | |
| (i) | (j) | Mean difference (i-j) | | st. error | sig.^a^ | | Mean difference (i-j) | | st. error | sig.^b^ | Mean difference (i-j) | st. error | sig.^b^ | Mean difference (i-j) | st. error | sig.^a^ | Mean difference (i-j) | st. error | sig.^b^ |
| Parents/relatives | Other adults/family home | 2.58260* | | 0.626 | **0.000** | | 7.76875* | | 0.897 | **0.000** | 7.00401* | 0.861 | **0.000** | 2.53543* | 0.971 | **0.025** | 1.69858* | 0.346 | **0.000** |
|  | HVB-hem | 3.26811* | | 0.752 | **0.000** | | 11.48094* | | 1.012 | **0.000** | 14.08420* | 1.136 | **0.000** | 6.25854* | 1.155 | **0.000** | 2.04536* | 0.508 | **0.000** |
| Other adults/ family home | Parents/relatives | -2.58260* | | 0.626 | **0.000** | | -7.76875* | | 0.897 | **0.000** | -7.00401* | 0.861 | **0.000** | -2.53543* | 0.971 | **0.025** | -1.69858* | 0.346 | **0.000** |
|  | HVB-hem | 0.686 | | 0.947 | 0.750 | | 3.71219* | | 1.293 | **0.013** | 7.08019* | 1.380 | **0.000** | 3.72311* | 1.461 | **0.029** | 0.347 | 0.601 | 0.918 |
| *Mean difference significant at 0.05 level | | | ^a^Tukey post-hoc test | | | ^b^Tamhane post-hoc test | | | | |  |  |  |  |  |  |  |  |  |
|  |  |  | |  |  | |  | |  |  |  |  |  |  |  |  |  |  |  |
| **Family economy - test proves homogeneity of variance for SS, so use Tukey post-hoc and Tamhane for PH, PW, PA, SC:** | | | | | | | | | | |  |  |  |  |  |  |  |  |  |
|  |  | Physical wellbeing (PH) | | | | | Psychological wellbeing (PW) | | | | Parent relations and autonomy (PA) | | | Social support and peers (SS) | | | School environment (SC) | | |
| (i) | (j) | Mean difference (i-j) | | st. error | sig.^b^ | | Mean difference (i-j) | | st. error | sig.^b^ | Mean difference (i-j) | st. error | sig.^b^ | Mean difference (i-j) | st. error | sig.^a^ | Mean difference (i-j) | st. error | sig.^b^ |
| Good | Average | 1.89750* | | 0.341 | **0.000** | | 4.79465* | | 0.559 | **0.000** | 3.75526* | 0.515 | **0.000** | 4.20000* | 0.529 | **0.000** | .50131* | 0.182 | **0.018** |
|  | Poor | 4.56287* | | 0.477 | **0.000** | | 11.80548* | | 0.667 | **0.000** | 11.82944* | 0.625 | **0.000** | 8.90809* | 0.679 | **0.000** | 1.83021* | 0.256 | **0.000** |
| Average | Good | -1.89750* | | 0.341 | **0.000** | | -4.79465* | | 0.559 | **0.000** | -3.75526* | 0.515 | **0.000** | -4.20000* | 0.529 | **0.000** | -.50131* | 0.182 | **0.018** |
|  | Poor | 2.66537* | | 0.478 | **0.000** | | 7.01083* | | 0.648 | **0.000** | 8.07418* | 0.607 | **0.000** | 4.70809* | 0.668 | **0.000** | 1.32889* | 0.255 | **0.000** |
| *Mean difference significant at 0.05 level | | | ^a^Tukey post-hoc test | | | | | ^b^Tamhane post-hoc test | | |  |  |  |  |  |  |  |  |  |
|  |  |  | |  |  | |  | |  |  |  |  |  |  |  |  |  |  |  |
| **Country of birth - test proves homogeneity of variance for none, so use Tamhane for all:** | | | | | | | | |  |  |  |  |  |  |  |  |  |  |  |
|  |  | Physical wellbeing (PH) | | | | | Psychological wellbeing (PW) | | | | Parent relations and autonomy (PA) | | | Social support and peers (SS) | | | School environment (SC) | | |
| (i) | (j) | Mean difference (i-j) | | st. error | sig.^b^ | | Mean difference (i-j) | | st. error | sig.^b^ | Mean difference (i-j) | st. error | sig.^b^ | Mean difference (i-j) | st. error | sig.^b^ | Mean difference (i-j) | st. error | sig.^b^ |
| Afghanistan | Iraq | -2.72111* | | 0.497 | **0.000** | | -7.14416* | | 0.768 | **0.000** | -7.60325* | 0.717 | **0.000** | -5.42653* | 0.762 | **0.000** | -1.86911* | 0.255 | **0.000** |
|  | Syria | -2.37904* | | 0.366 | **0.000** | | -6.50373* | | 0.569 | **0.000** | -7.27889* | 0.526 | **0.000** | -5.70803* | 0.549 | **0.000** | -1.69562* | 0.202 | **0.000** |
| Iraq | Afghanistan | 2.72111* | | 0.497 | **0.000** | | 7.14416* | | 0.768 | **0.000** | 7.60325* | 0.717 | **0.000** | 5.42653* | 0.762 | **0.000** | 1.86911* | 0.255 | **0.000** |
|  | Syria | 0.342 | | 0.448 | 0.829 | | 0.640 | | 0.710 | 0.747 | 0.324 | 0.661 | 0.947 | -0.281 | 0.693 | 0.969 | 0.173 | 0.219 | 0.812 |
| *Mean difference significant at 0.05 level | | | ^b^Tamhane post-hoc test | | | | | |  |  |  |  |  |  |  |  |  |  |  |

Online Resource 2. Sum score scale comparison between refugees 12-18 years and EU norms


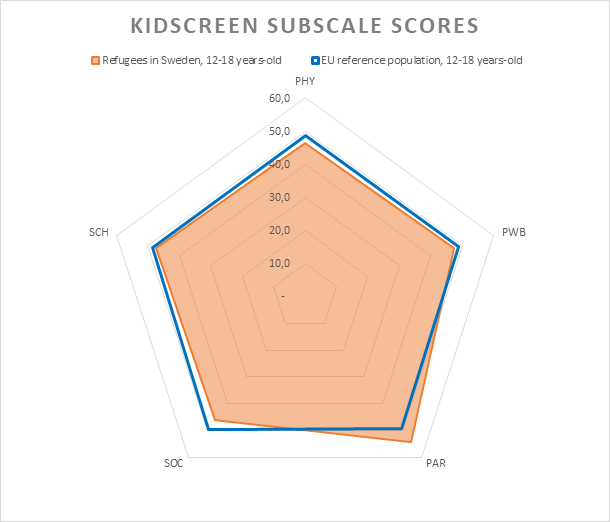


PHY: physical wellbeing, PWB: psychological wellbeing, PAR: parent/caregiver relations and autonomy, SOC: social support and peers, SCH: school environment
